# Resting-state fMRI study of brain activation using low-intensity repetitive transcranial magnetic stimulation in rats

**DOI:** 10.1038/s41598-018-24951-6

**Published:** 2018-04-30

**Authors:** Bhedita J. Seewoo, Kirk W. Feindel, Sarah J. Etherington, Jennifer Rodger

**Affiliations:** 1 Experimental and Regenerative Neurosciences, School of Biological Sciences, The University of Western Australia, Perth, WA Australia; 2 Centre for Microscopy, Characterisation and Analysis, Research Infrastructure Centres, The University of Western Australia, Perth, WA Australia; 3 School of Veterinary and Life Sciences, Murdoch University, Perth, WA Australia; 4 School of Biomedical Sciences, The University of Western Australia, Perth, WA Australia; 5 Brain Plasticity Group, Perron Institute for Neurological and Translational Research, Perth, WA Australia

## Abstract

Repetitive transcranial magnetic stimulation (rTMS) is a non-invasive neuromodulation technique used to treat many neuropsychiatric conditions. However, the mechanisms underlying its mode of action are still unclear. This is the first rodent study using resting-state functional MRI (rs-fMRI) to examine low-intensity (LI) rTMS effects, in an effort to provide a direct means of comparison between rodent and human studies. Using anaesthetised Sprague-Dawley rats, rs-fMRI data were acquired before and after control or LI-rTMS at 1 Hz, 10 Hz, continuous theta burst stimulation (cTBS) or biomimetic high-frequency stimulation (BHFS). Independent component analysis revealed LI-rTMS-induced changes in the resting-state networks (RSN): (i) in the somatosensory cortex, the synchrony of resting activity decreased ipsilaterally following 10 Hz and bilaterally following 1 Hz stimulation and BHFS, and increased ipsilaterally following cTBS; (ii) the motor cortex showed bilateral changes following 1 Hz and 10 Hz stimulation, a contralateral decrease in synchrony following BHFS, and an ipsilateral increase following cTBS; and (iii) hippocampal synchrony decreased ipsilaterally following 10 Hz, and bilaterally following 1 Hz stimulation and BHFS. The present findings demonstrate that LI-rTMS modulates functional links within the rat RSN with frequency-specific outcomes, and the observed changes are similar to those described in humans following rTMS.

## Introduction

Repetitive transcranial magnetic stimulation (rTMS) has been shown to have therapeutic potential for a range of psychiatric conditions, including unipolar^1,2^ and bipolar depression^1^, schizophrenia^3^, obsessive-compulsive disorder^4^ and post-traumatic stress disorder^5^ as well as neurological conditions such as Parkinson’s disease^6^, dystonia^7^, tinnitus^8^, epilepsy^9^ and stroke^10^. rTMS has also shown promising results in the treatment of pain syndromes such as migraine^11^ and chronic pain^12^. Even though rTMS is being used in a clinical setting and clinical trials are abundant, little is known about the mechanisms underlying its efficacy^13^. This knowledge gap is in part because human studies use mostly non-invasive methods such as functional magnetic resonance imaging (fMRI), TMS and behaviour to investigate the effects of rTMS while animal studies mostly use invasive methods.

Resting-state fMRI (rs-fMRI) is used to detect functionally linked brain regions whose patterns of spontaneous blood oxygenation level dependent contrast fluctuations are temporally correlated when the subject is at rest, that is, when no specific stimulus or task is presented^14^. Brain regions with coherent spontaneous fluctuations in activity form an organised network called the resting-state network (RSN)^14^. The default mode network (DMN) is one of the RSNs with a synchronised activity pattern. The DMN has been associated with cognitive performance and is thought to play an important role in neuroplasticity through the consolidation and maintenance of brain function^15^. rTMS is able to modulate the resting-state activity of the brain and DMN plasticity is sensitive to rTMS in humans but the direction (increase or decrease in activity) and extent of this modulation depend on the rTMS protocol used^16–20^.

Interleaving rs-fMRI and rTMS has opened doors to many possibilities in the clinical setting as rs-fMRI allows for direct visualisation of rTMS-induced effects in the brain. However, there have been no reports of rodent studies using those same techniques^21^. Because rodents are widely used as preclinical models of various neuropsychiatric disorders, a thorough understanding of how rTMS affects the rodent DMN is of particular importance for both interpreting rodent rs-fMRI data and translating findings between animal models and humans. The present study aimed to investigate whether low intensity (LI) rTMS, which allows focal application of low intensity pulsed magnetic fields to one hemisphere of the brain in rodents, alters the strength or the spatial distribution, or both, of the RSN activity in rats. We used LI-rTMS because of its relatively high focality compared to rTMS delivered at high intensity using human rTMS equipment^22^, and LI-rTMS has previously been shown to induce cellular and molecular changes in rodent brains^23–25^. We show that LI-rTMS alters the resting-state activity of neurons directly at the site of stimulation as well as in brain regions that have direct connections with the site of stimulation. Moreover, the magnitude and pattern of the change in resting-state neuronal activity depend on the frequency and pattern of LI-rTMS. Therefore, these findings have relevance for establishing a direct comparison between human and animal models in terms of how magnetic fields affect resting neuronal activity and ultimately, may prove helpful in the development of evidence-based rTMS treatment protocols to modify functional connectivity abnormalities.

## Methods

### Ethics statement

Experimental procedures were approved by the UWA Animal Ethics Committee (RA/3/100/1430) and Murdoch Animal Ethics Committee (IRMA2848/16) and conducted in accordance with National Health and Medical Research Council Australian code for the care and use of animals for scientific purposes.

### Animals

Six adult male Sprague Dawley rats between six and eight weeks old (150–250 g) were sourced from the Animal Resources Centre (Canning Vale, WA, Australia). They were maintained in a temperature-controlled animal care facility on a 12-hour light-dark cycle with food and water *ad libitum* with one-week habituation before the start of experiments.

### Experimental Protocol

**Figure 1:**
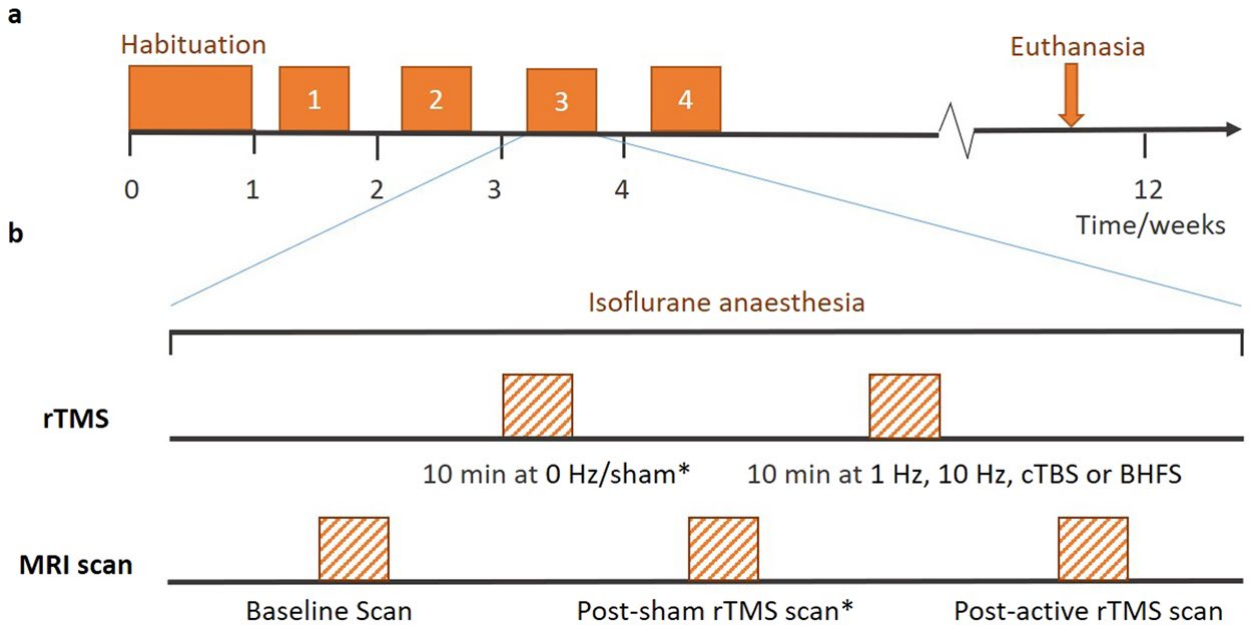
Experimental protocol. (**a**) Timeline for a single rat from the time of its arrival. The experiment consisted of a habituation period followed by four sessions of fMRI-LI-rTMS-fMRI. Sessions one to four were the same, except for the frequency of LI-rTMS used and whether 0 Hz/sham stimulation preceded actual stimulation. (**b**) Protocol for a single LI-rTMS/rs-fMRI session. During each session, baseline rs-fMRI data were acquired after which stimulation using a specific protocol (1 Hz, 10 Hz, BHFS or cTBS) was delivered. A post-procedure rs-fMRI scan was then carried out. *Sham stimulation and post-sham LI-rTMS scan were carried out only once for each animal. The session during which sham stimulation was delivered and the frequency at which active LI-rTMS was delivered during the same session was randomly determined.

During each session, the animal was first anaesthetised using isoflurane gas and was kept under isoflurane anaesthesia throughout the experiment. Each rat received LI-rTMS for 10 minutes to the right hemisphere with one of four stimulation protocols (1 Hz, 10 Hz, combined theta burst stimulation (cTBS) and biomimetic high-frequency stimulation (BHFS), randomised order) in the morning once a week for four weeks (Fig. 1). The timing of the experiments was dependent on the availability of imaging equipment but at least one week was allowed between sessions to allow for any effect of LI-rTMS to subside^26^. Rs-fMRI scans were performed immediately before and after the stimulation session. In addition, sham/0 Hz stimulation was delivered on a randomly determined day, prior to completion of the randomly selected stimulation protocol for that day. Sham stimulation and post-sham rs-fMRI scan were carried out only once for each animal. Animals were kept for up to 12 weeks and were euthanised after the last rTMS/fMRI session using carbon dioxide asphyxiation.

### Animal preparation for MRI

Once fully anaesthetised in an induction chamber (4% isoflurane in 100% medical oxygen, 2 L/min), the animal was transferred to a heated imaging cradle and anaesthesia was maintained with a nose cone (1–2.5% isoflurane in 100% medical oxygen, 1 L/min). Body temperature, respiratory rate, heart rate, and blood oxygen saturation were monitored using a PC-SAM Small Animal Monitor (SA instruments Inc., 1030 System).

### rTMS procedure

LI-rTMS was delivered using a custom-built round coil (8 mm inside diameter, 16.2 mm outside diameter, 10 mm thickness, 0.25 mm copper wire, 6.1 Ω resistance, 462 turns) placed on the right side of the rat brain next to the right ear^27^. The coil and pulse generator (Model EXLAB606, Serial Number 00003) were designed and built by Global Energy Medicine (Perth, WA, Australia). The device is described in detail in Grehl, *et al*.^27^. Each stimulation protocol (1 Hz, 10 Hz, cTBS or BHFS) had a specific pre-programmed card such that when inserted into the generator, the phase transitions were triggered automatically (see Supplementary Table S1). Limitations of the equipment meant that intermittent theta burst stimulation (iTBS), commonly used in humans^28^, could not be delivered (the maximum pulse interval was 1 s).

### Magnetic field measurements

The magnetic field generated by the coil was measured using a gaussmeter connected to an oscilloscope. The transverse Hall probe was fixed to a stereotaxic frame and manipulated around the coil. Measurements were taken in the perpendicular (xy) and parallel (z) axes relative to the main axis of the coil. Due to the axial symmetry of the circular coil, measurements in the x axis also represent the y axis and are therefore referred to as xy. The Hall probe head was positioned near the centre of the coil, at the edge and half-way between the centre and the edge of the coil (xy, z = 0 mm). At each of these three positions on the coil, the probe was repositioned at 1 mm increments away from the coil surface to a maximum distance of 10 mm (zmax = +10 mm) to determine the intensities at which different parts of the brain received the stimulation. The monophasic pulse generated an intensity of approximately 13 mT at the surface of the cortex, which is below motor threshold^27^.

### MRI data acquisition

All MR images were acquired with a Bruker Biospec 94/30 small animal MRI system operating at 9.4 T (400 MHz, H-1), with an Avance III HD console, BGA-12SHP imaging gradients, an 86 mm (inner diameter) volume transmit coil and a rat brain surface quadrature receive coil. ParaVision 6.0.1 software was used to control the scanner and set the experimental tasks. Following a tri-plane scan to determine the position of the rat brain, high-resolution T2-weighted coronal images were acquired using a multi-slice 2D RARE (Rapid Acquisition with Relaxation Enhancement) sequence with fat suppression from 21 × 1-mm-thick interlaced slices with slice gap of 0.05 mm and: field-of-view (FOV) = 28.0 mm × 28.0 mm; matrix size (MTX) = 280 × 280; 0.1 mm × 0.1 mm in-plane pixel size; repetition time (TR) = 2500 ms; echo time (TE) = 33 ms; RARE factor = 8; echo spacing = 11 ms; number of averages (NA) = 2; number of dummy scans (DS) = 2; flip angle (α) = 90°; receiver bandwidth (BW) = 34722.2 Hz; and scan time = 2 min 55 s. Prior to acquiring the fMRI data, B0 shimming was completed for a region of interest covering the brain using the Bruker Mapshim routine. T2^*^ weighted fMRI images were acquired using a single-shot echo planar imaging (EPI) sequence with: FOV = 28.2 mm × 21.0 mm; MTX = 94 × 70; 0.3 mm × 0.3 mm in-plane pixel size; TR = 1500 ms; TE = 11 ms; NA = 1; DS = 8; 300 repetitions; BW = 326087.0 Hz; 58/70 partial Fourier acquisition in the phase encode dimension; and scan time = 7 min 30 s. All radio frequency pulse shapes were calculated automatically using the Shinnar-Le Roux algorithm^29–33^. The images acquired and analysed during the study are available from the corresponding author on reasonable request.

### Image processing

Most of the pre-processing and analyses were performed using FSL v5.0.9 (Functional MRI of the Brain (FMRIB) Software Library)^34^. The Bruker data was exported from ParaVision 6.0.1 into DICOM (Digital Imaging and Communications in Medicine) format^35^ (http://dicom.nema.org/) and then converted into NifTI (Neuroimaging Informatics Technology Initiative, https://nifti.nimh.nih.gov/) using the dcm2nii converter (64-bit Linux version 5 May 2016)^36^. Pre-processing of fMRI data included: (i) upscaling the voxel sizes by a factor of 10^37^; (ii) motion correction using FSL/MCFLIRT (Linear Image Registration Tool with Motion Correction)^38^ to spatially realign the functional images to the middle volume of a serial acquisition; and (iii) reorienting the brain into left-anterior-superior (LAS) axes (radiological view). Intracranial binary brain masks were created manually using ITK-SNAP 3.4.0^39^ (www.itksnap.org) for each functional and anatomical dataset and were used to extract the brain using the flsmaths tool. Post-stimulation images were co-registered to the baseline fMRI image using 6 parameter rigid body registration with the default correlation ratio cost metric in the FSL/FLIRT (Linear Image Registration Tool)^38,40^.

Single-session independent component analysis (ICA) was carried out for each brain-extracted dataset in FSL/MELODIC (Multivariate Exploratory Linear Decomposition into Independent Components)^41^ with the Gaussian kernel filter set to a full-width half maximum (FWHM) of 5 mm and a temporal high pass filter cut-off of 100 s. Based on the characteristics (spatial, temporal and frequency domains) of the components from ICA, they were then manually labelled as ‘signal’ or ‘noise’ and the data was ‘cleaned’ by removing the noise components using the fsl_regfilt command on the filtered data from MELODIC. The pre- and post-stimulation de-noised fMRI images for each session were then co-registered to their respective T2-weighted images using six parameter rigid body registration^42^. To facilitate automated processing, the images were normalized to a Sprague Dawley brain atlas^43–45^ using FLIRT with nine degrees of freedom ‘traditional’ registration. The atlas was first down-sampled by a factor of eight to better match the voxel size of the 4D functional data. All subsequent analyses were conducted in the atlas standard space.

### Image analysis

**Figure 2:**
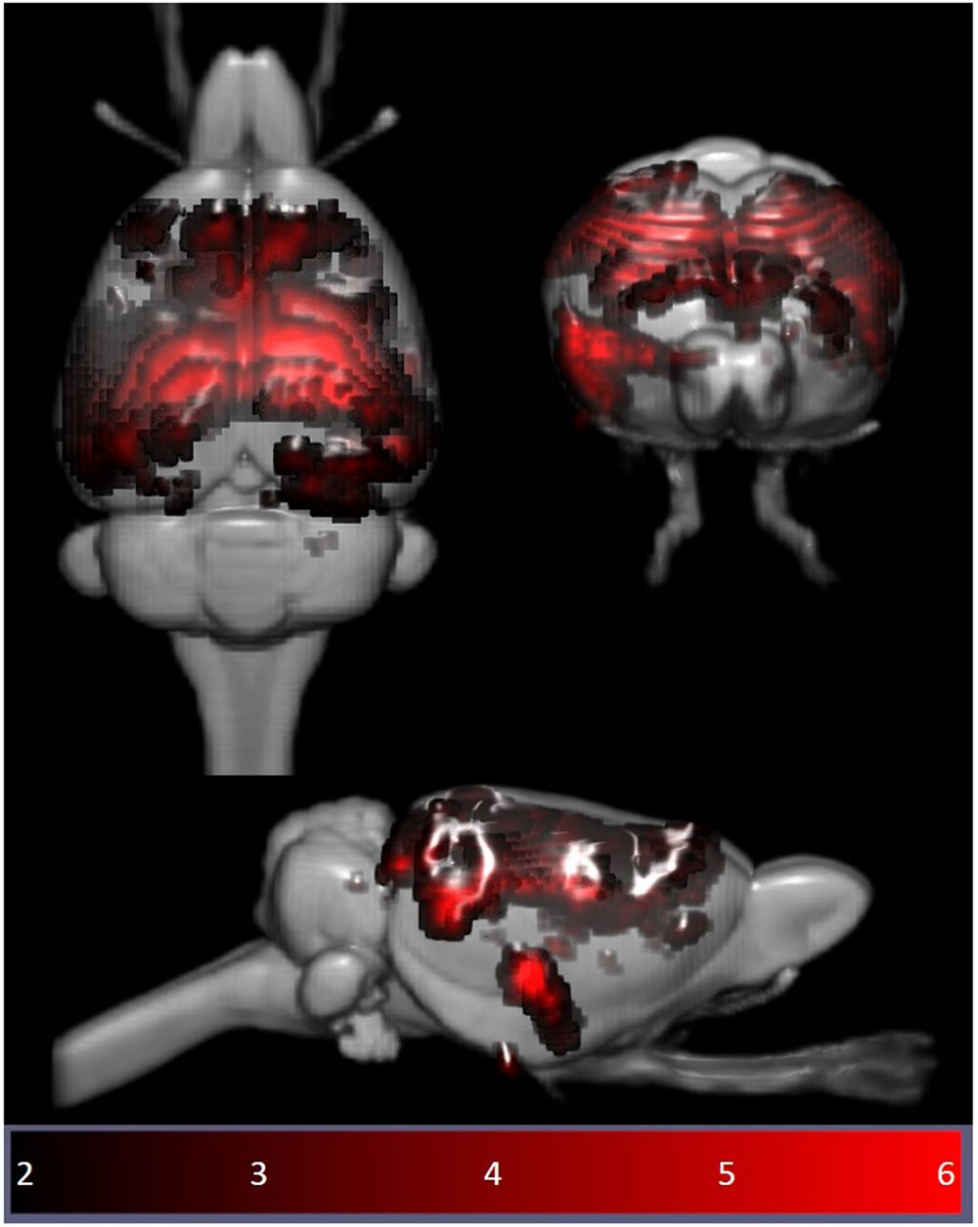
Independent component maps of pre-stimulation rs-fMRI group overlaid on 3D-rendered standard Sprague Dawley brain atlas. The figure shows the superior view (top left), anterior view (top right) and lateral view (bottom) of the chosen six non-artefactual independent components from the group-ICA. The spatial colour-coded z-maps of these components are overlaid on the brain atlas (down-sampled by a factor of eight). A higher z-score (bright red) represents a higher correlation between the time course of that voxel and the mean time course of the components. Colour bar indicates z-scores (n = 24, thresholded at z > 2, uncorrected p-value < 0.0455 for a two-tailed hypothesis).

**Figure 3:**
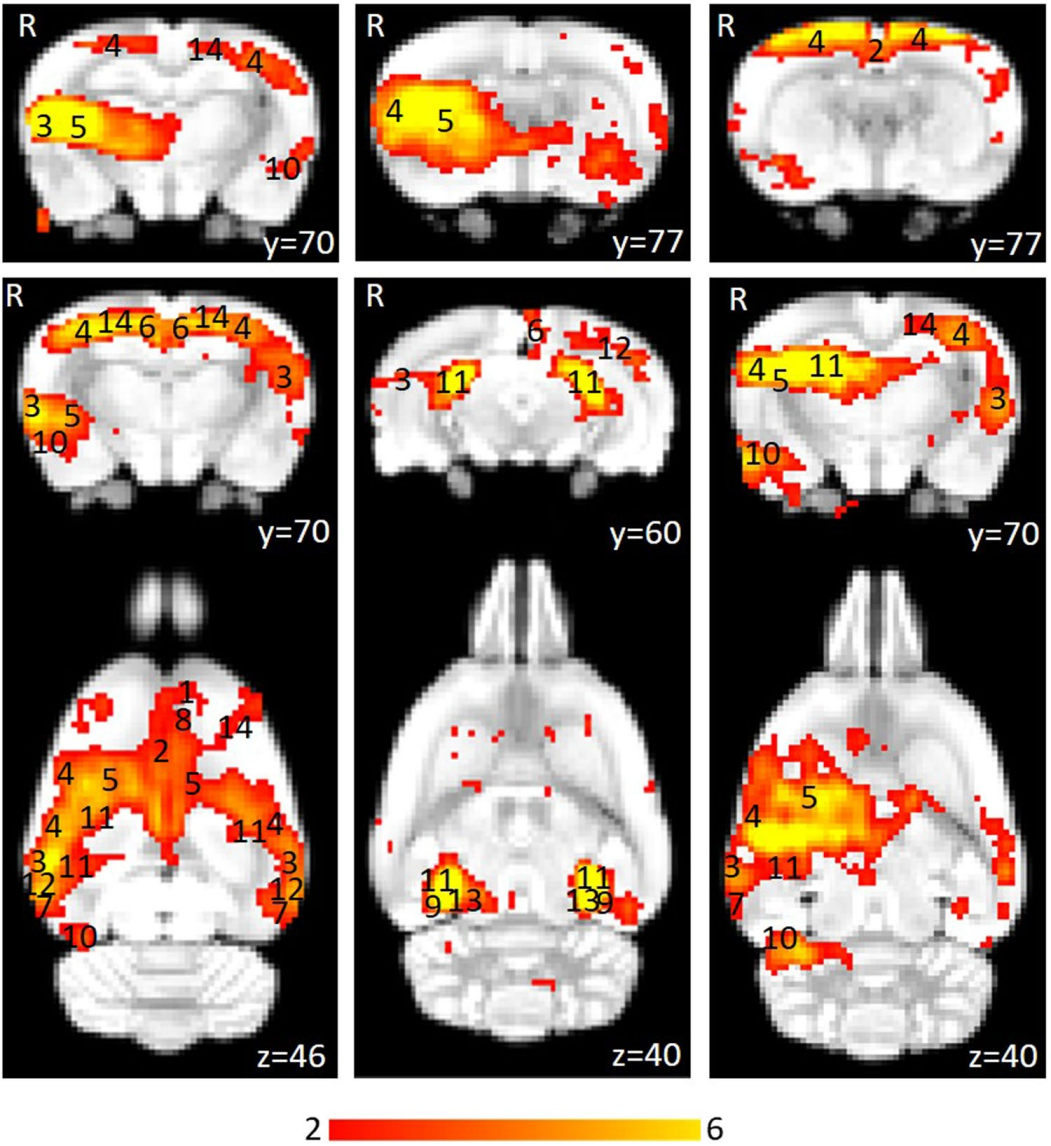
Thresholded independent component spatial maps showing the resting-state network in the pre-stimulation rs-fMRI group-ICA dataset. The figure shows six independent components from the group-ICA, three coronal slices and three coronal slices with their corresponding axial slices. The spatial colour-coded z-maps of these components are overlaid on the brain atlas (down-sampled by a factor of eight). A higher z-score (yellow) represents a higher correlation between the time course of that voxel and the mean time course of this component. Significant clusters within the components include various brain regions: 1, orbital cortex; 2, cingulate cortex; 3, auditory cortex; 4, somatosensory cortex; 5, striatum/caudate putamen; 6, retrosplenial cortex; 7, temporal association cortex; 8, prelimbic cortex; 9, parasubiculum; 10, entorhinal cortex; 11, hippocampus; 12, visual cortex; 13, inferior colliculus; 14, motor cortex. R denotes right hemisphere. x, y, z refer to the coordinates in standard Sprague Dawley template space. Colour bar indicates z-scores (n = 24, thresholded at z > 2, uncorrected p-value < 0.0455 for a two-tailed hypothesis).

Multi-subject temporal concatenation group-ICA was performed to determine group differences by comparing pre- and post-stimulation fMRI images. The ICA algorithm was restricted to 15 components on the basis of other rs-fMRI studies in rodents^46,47^ and was performed with the MELODIC toolbox. Group-ICA on the pre-stimulation datasets was also carried out with 30 components to determine whether 15 components were sufficient to identify the DMN. Given the limited sample size and the novelty of the parameters of interest, we report the results based on a cluster-forming threshold of z > 2, corresponding to an uncorrected p-value < 0.0455 for a two-tailed hypothesis. The group-ICA components for the pre-stimulation group (z > 2) were visually inspected, and the DMN identified (Fig. 2) based on the spatial patterns in reference to known anatomical and functional locations using a rat brain atlas^48^. After identifying the DMN (Fig. 3), pre- and post-rTMS homologous ICA components were visually compared to determine the effect of LI-rTMS on the DMN.

**Figure 4:**
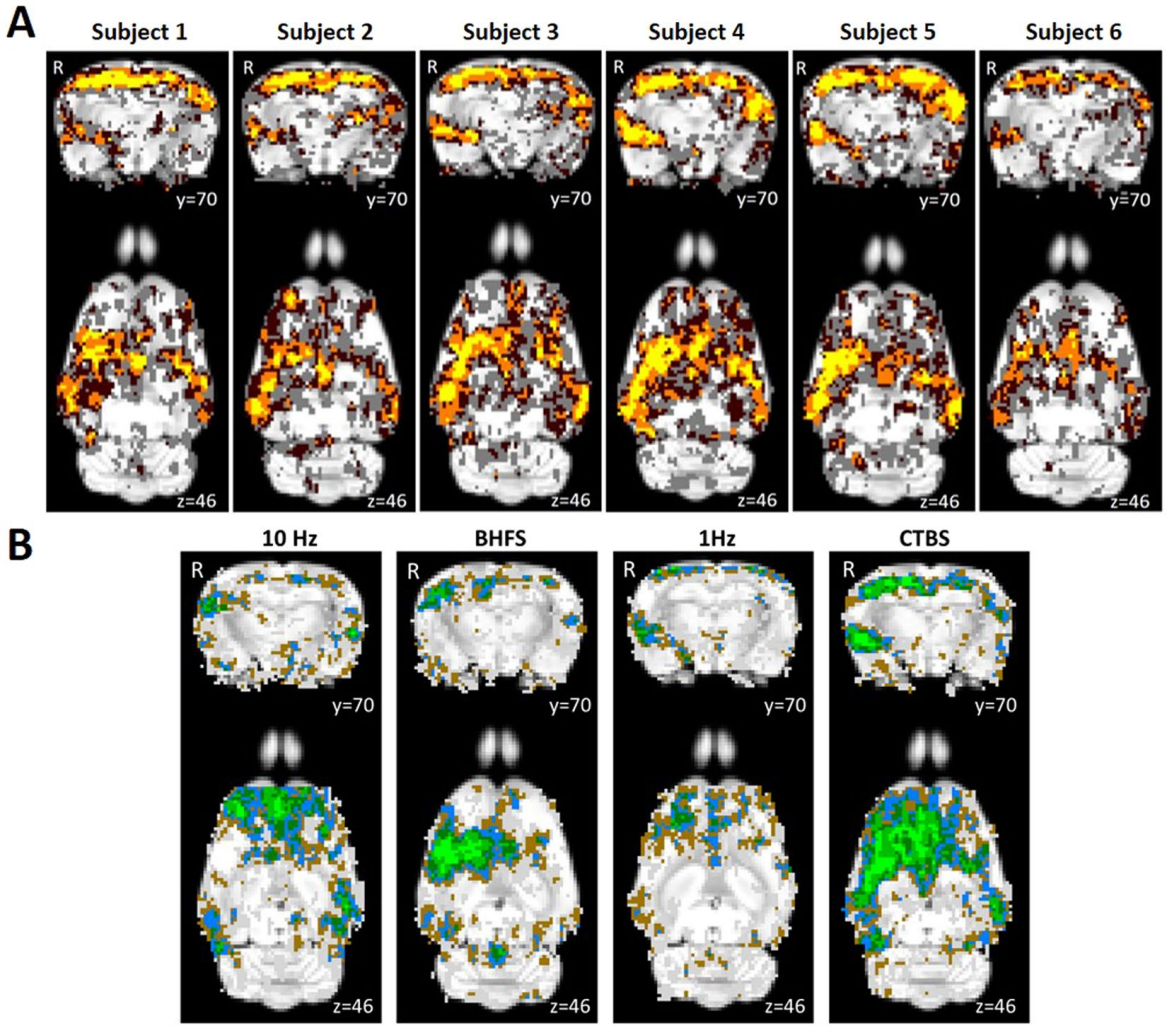
Reproducibility between sessions and between subjects of a representative group-ICA component. (**A**) Pre-stimulation session cumulative score maps of six subjects over the four different time points overlaid on the Sprague Dawley rat brain atlas (down-sampled by a factor of eight). x, y and z refer to the coordinates in standard Sprague Dawley template space. Colour code: voxels with z-value greater than 2 (uncorrected p-value < 0.0455 for a two-tailed hypothesis) for: one session, grey; two sessions, dark brown; three sessions, orange; four sessions, yellow. (**B**) Animal cumulative score maps of six subjects following stimulation at 10 Hz, BHFS, 1 Hz and cTBS overlaid on the Sprague Dawley rat brain atlas (down-sampled by a factor of eight). x, y and z refer to the coordinates in standard Sprague Dawley template space. Colour code: voxels with z-value greater than 2 (uncorrected p-value < 0.0455 for a two-tailed hypothesis) for: one animal, grey; two animals, brown; three animals, blue; four animals, dark green; five animals, green; six animals, bright green.

As data was acquired for each animal at four different timepoints, the reproducibility of the group-ICA results over time and between subjects was also investigated. The pre-stimulation datasets were thresholded to a z-score higher than 2, binarised and then summed to give cumulative reproducibility maps for each subject (Fig. 4). The regions where the voxels have a z-score of 2 or higher for one, two, three or four sessions are shown in different colours.

## Results and Discussion

Rs-fMRI studies in rodents have previously shown that rodents possess a DMN similar to humans despite the distinct evolutionary paths between rodent and primate brains^49^. In this study, we compared spontaneous activity in the brain at rest before and after the animals received active or sham LI-rTMS over the right hemisphere. The RSN in the rat brain was inferred based on synchronous fluctuations of the haemodynamic signals identified by ICA of pre-stimulation rs-fMRI data. ICA of post-stimulation rs-fMRI data showed that 10 Hz stimulation, BHFS and cTBS caused mostly ipsilateral changes in synchrony of resting activity while 1 Hz stimulation resulted in bilateral changes in synchrony, with the contralateral changes being more prominent than ipsilateral changes. When compared with results from rTMS/fMRI studies in humans, our findings suggest that repetitive transcranial magnetic stimulation, whether in the form of conventional high intensity rTMS in humans, or the lower intensity version LI-rTMS used here in rats, has similar effects on human and rat resting brain activity. Therefore, LI-rTMS/fMRI studies in animal models may be useful in refining clinical protocols for humans.

### Identification of resting-state brain networks

The pre-stimulation group data were analysed using the group-ICA algorithm, and the resting-state networks were identified (Fig. 2). The components obtained from MELODIC were overlaid on the rat brain template to which they were originally co-registered and the distribution of the synchronised voxels was investigated using the digital brain atlas labels. Based on visual inspection of the spatial map for each of the 15 components and the consistency of the spatial distribution with known neuroanatomical regions from the brain atlas, six non-artefactual circuits could be identified, which formed part of the putative DMN (Fig. 3). The remaining nine components were classified as noise (see examples in Supplementary Fig. S1).

The DMN in rats has previously been described as consisting of the orbital cortex^47,49–52^, cingulate cortex^46,47,49–52^, auditory cortex^49,51,52^, somatosensory cortex^51^, striatum/caudate putamen^51^, retrosplenial cortex^47,49–51^, temporal association cortex^47,49,52^, prelimbic cortex^47,50,52^, parasubiculum^47^, entorhinal cortex^47^, hippocampus^49,51,52^ and visual cortex^49,52^. Figure 3 provides the ICA components with clusters corresponding to these regions. The motor cortex and inferior colliculus, which form part of the RSN^46^ have also been identified.

In the pre-stimulation group, the ICA components forming the RSN showed bilateral symmetry in resting activity (Fig. 2). However, four of the chosen non-artefactual ICA components (Fig. 3) had spatially asymmetrical correlations between homologous brain regions. In some components, the homologous brain region was completely absent (no correlation at that particular time point) while in some components, the spatial extent of the clusters was larger in one hemisphere. The ‘dominant’ hemisphere with increased ipsilateral cluster size was the same between sessions and across all animals (Fig. 4). Coherent neuronal oscillations or spontaneous rhythmic activity are believed to show which brain regions are coupled for joint processing for a specific function, and the resulting hemodynamic responses are interpreted as functional connectivity between these areas^53^. MacDonald, *et al*.^54^ measured the oscillations of the auditory and somatosensory cortex in anaesthetised rats and found that the oscillations in the somatosensory cortex were stronger in one hemisphere. Other similar behavioural and electrophysiological studies have shown that homologous brain regions can function both unilaterally and bilaterally^53,55^. This functional ability is a possible explanation for the apparently stronger synchrony in resting-state activity unilaterally in some brain regions within those ICA components. For example, the first ICA component in Fig. 3 shows that there is a strong synchrony in the resting activity of the right auditory cortex (3) and right striatum (5), but there are no clusters for homologous brain regions in the left hemisphere. Such asymmetries in functional networks have previously been reported in resting-state network studies using ICA^47,56,57^. The unilateral components could represent stronger local connectivity, which could be both independent of, and synchronised with, the inter-hemispheric connectivity within the RSN.

Interestingly, some brain regions, including the auditory cortex (3) and striatum (5), show unilateral synchrony in some components and bilateral synchrony in others. A previous study using ICA to identify resting-state networks in rats also reported that functionally connected regions can split into separate components^57^. Similar observations were made when the group-ICA algorithm was limited to 30 components instead of 15. The observed z-score ICA spatial maps of 30-component analysis were very similar to the 15-component analysis but the increased number of components caused components belonging to the same functional networks to split into different components previously identified in Hutchison, *et al*.^57^. The resting activity of these brain regions split into different components based on higher local synchrony in activity^46,47^. Overall, these results confirm that the group-ICA algorithm can cause homologous brain regions within the DMN to appear in separate components and the extent of this is dependent on the strength of bilateral synchrony and the total number of components^46,47^.

### Reproducibility over time and between subjects

Each animal was scanned at four timepoints, enabling the investigation of the reproducibility of the group-ICA results over time and between subjects using pre-stimulation rs-fMRI data (Fig. 4A). The reproducibility maps illustrate that the middle part of the clusters show overlap for all or at least three timepoints while the border of the clusters represents data from only one or two time points. Most of the scatter and single-voxel correlations come from single sessions (shown in grey). This indicates that the central resting-state activity is reproducible, even when the rs-fMRI data acquisition is separated by a week or more.

Comparing the session cumulative maps between subjects, shows that the same pattern is seen in each of the six animals. Similar to the intersession reproducibility, the central part of the representative component for the animal cumulative maps (Fig. 4B) overlaps for more animals, while the voxels towards the border tend to represent data from single animals. This shows that the post-stimulation rs-fMRI data are reproducible between subjects as well.

Although our study did not address how long LI-rTMS effects persist after stimulation, the high reproducibility of baseline scans in the same animals a week apart suggest that any effect of stimulation has subsided. This is in line with studies in humans suggesting that rTMS effects are transient, lasting less than an hour. Future studies can take advantage of the longitudinal opportunities of rs-fMRI to study the duration of LI-rTMS effects at short timescales of hours to days.

### Effects of LI-rTMS on resting-state brain activity

**Figure 5:**
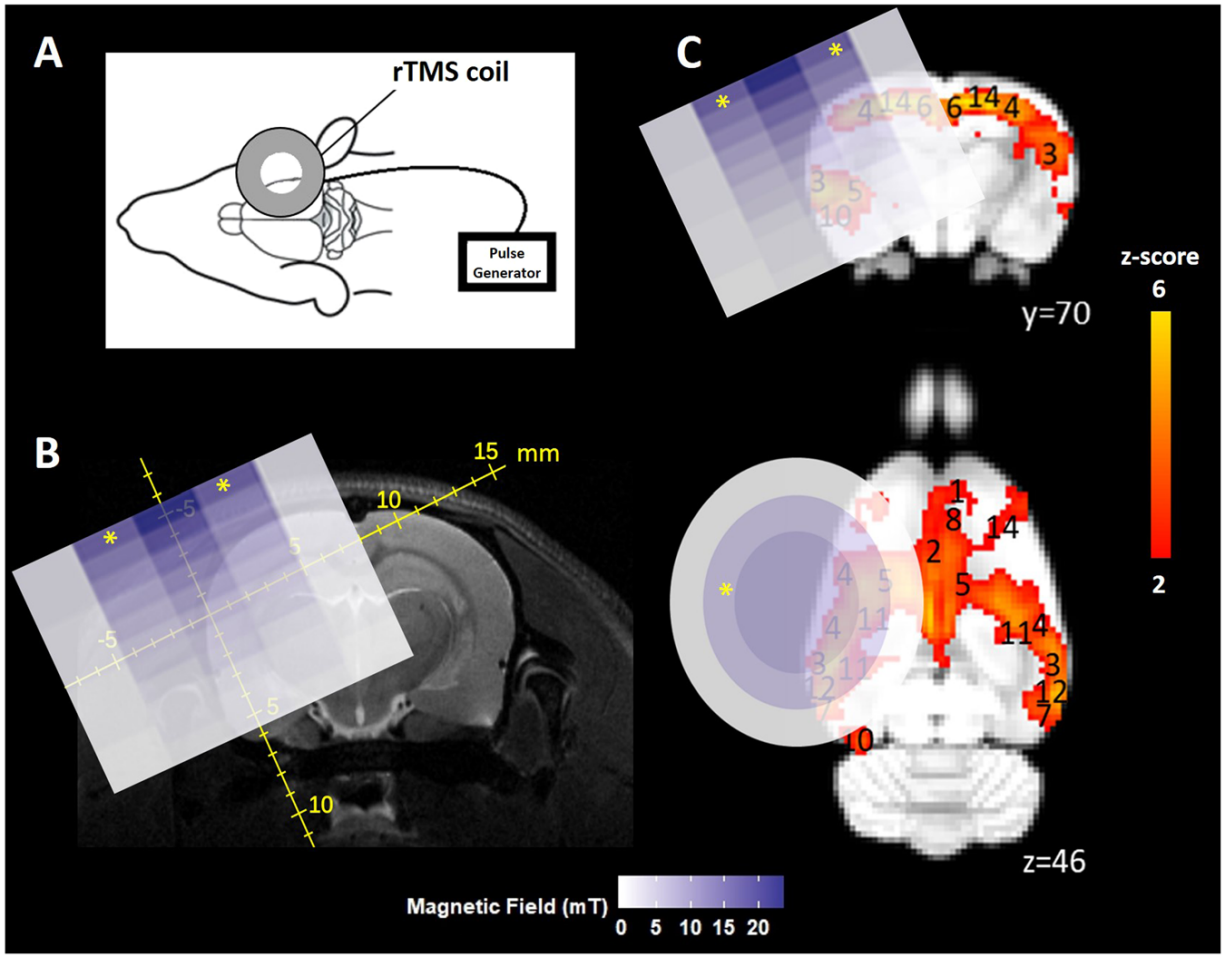
Coil position and magnetic field. (**A**) Coil position relative to rat head and brain. (**B**) 2D representation of the magnetic field induced by the LI-rTMS coil superimposed on a representative raw T2-weighted brain image with scale in mm. Measurements were taken on a hall device at 1 mm increments. (**C**) 2D representation of the magnetic field induced by the LI-rTMS coil superimposed on colour-coded coronal and axial slices for a representative pre-stimulation group-ICA component overlaid on the Sprague Dawley brain template (down-sampled by a factor of eight). White-blue colour bar indicates magnetic field intensities. Yellow-red colour bar indicates z-scores (n = 24, thresholded at z > 2, uncorrected p-value < 0.0455 for a two-tailed hypothesis). x, y, z refer to the coordinates in standard Sprague Dawley template space.“*” indicates the zone where electric field is induced.

**Figure 6:**
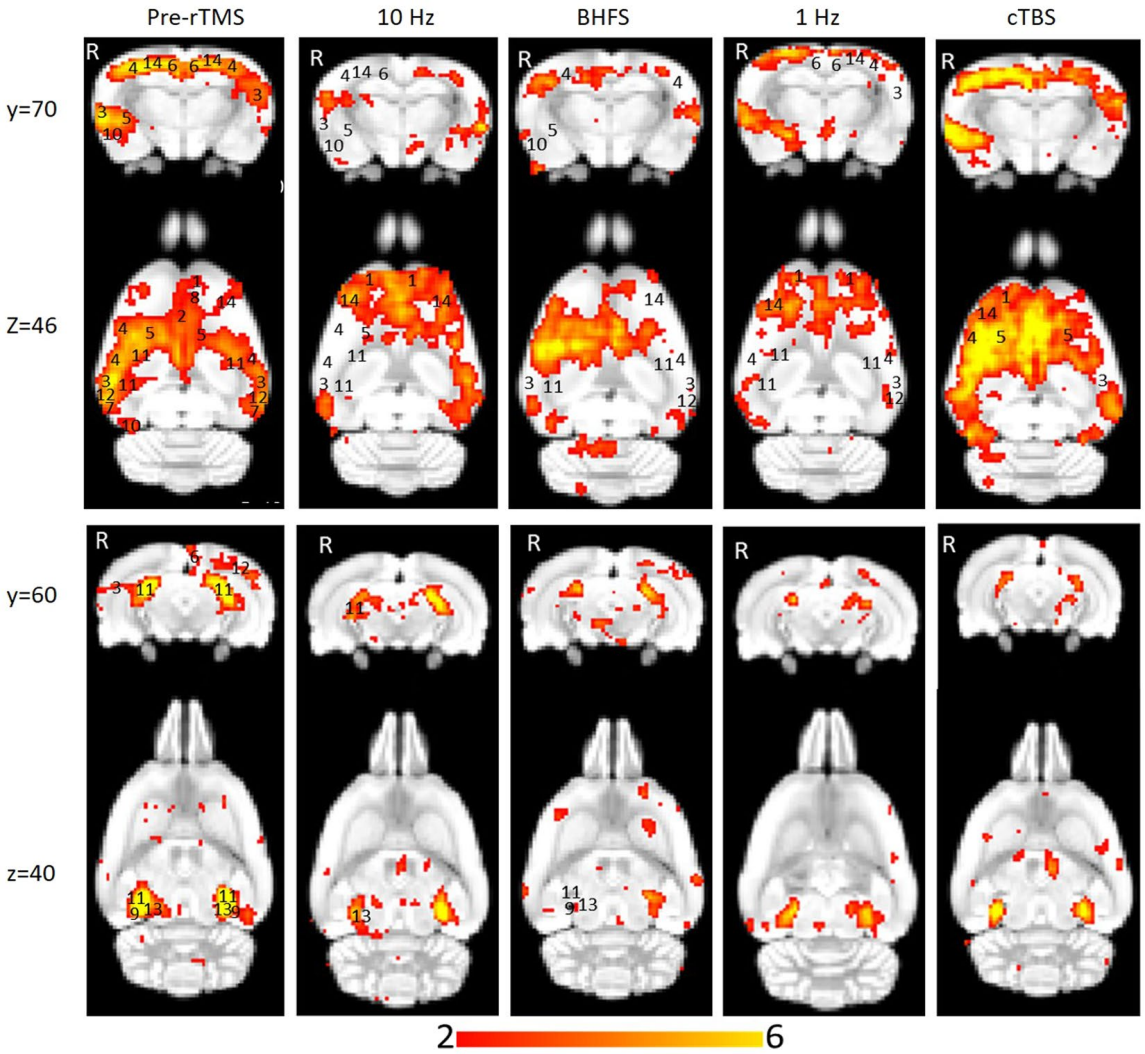
Homologous group-ICA components showing synchronised resting-state neuronal activity of isoflurane-anaesthetized rats before and after LI-rTMS at four frequencies: 10 Hz, BHFS, 1 Hz and cTBS. The post-stimulation colour-coded z-maps were derived from group-ICA on six animals and were overlaid on the Sprague Dawley brain template (down-sampled by a factor of eight). Coronal and axial slices for two representative ICA components are shown before (left) and after (right) stimulation at each of the four LI-rTMS protocols. A higher z-score (yellow) represents a higher correlation between the time course of that voxel and the mean time course of this component. Significant clusters within the components include various brain regions: 1, orbital cortex; 2, cingulate cortex; 3, auditory cortex; 4, somatosensory cortex; 5, striatum/caudate putamen; 6, retrosplenial cortex; 7, temporal association cortex; 8, prelimbic cortex; 9, parasubiculum; 10, entorhinal cortex; 11, hippocampus; 12, visual cortex; 13, inferior colliculus; 14, motor cortex. R denotes right hemisphere. x, y, z refer to the coordinates in standard Sprague Dawley template space. Colour bar indicates z-scores (thresholded at z > 2, uncorrected p-value < 0.0455 for a two-tailed hypothesis).

LI-rTMS was delivered to the right brain hemisphere (Fig. 5) with one of four stimulation protocols (1 Hz, 10 Hz, cTBS and BHFS) and group-ICA components for each post-stimulation dataset were compared to the non-artefactual components identified in the pre-stimulation group to investigate the effect of LI-rTMS. Changes in synchrony of resting activity are reported only for those changes involving whole brain regions, as identified in the atlas. There were no changes in any of the DMN components after sham stimulation. However, there were clear changes in the synchronised activity following active LI-rTMS (Fig. 6). Both excitatory frequencies (10 Hz and BHFS) displayed more noticeable ipsilateral changes in the strength of correlation within the DMN. The inhibitory frequency, 1 Hz, showed bilateral changes in most components, although there were more contralateral than ipsilateral changes in the synchronised activity of brain regions. cTBS caused mostly ipsilateral increases in synchrony, and the effects were not as widespread as the other LI-rTMS protocols (See Supplementary Table S2).

A change in the synchrony of resting-state activity in a specific brain region can be related to either an increase or a decrease in activity of that area compared to other brain regions within the same network. A decrease in synchrony, for example, does not necessarily mean a decrease in activity. In human rTMS, high-frequency stimulation ( ≥5 Hz) mostly causes an increase in cortical excitability while 1 Hz and cTBS are predominantly inhibitory frequencies, causing a decrease in cortical excitability^58,59^. However, excitability changes following LI-rTMS are not characterised to a sufficient degree to allow unequivocal interpretation of our rs-fMRI results. Nonetheless, early indications are that LI-rTMS may induce similar excitability changes to human (high intensity) rTMS, despite different magnetic field intensities^25^. Therefore, we discuss the changes in synchrony we observed in rats in the context of excitability changes reported in human rTMS literature, but do so with caution. Herein, the discussion focuses on the effect of LI-rTMS on the somatosensory cortex, motor cortex and hippocampus.

#### Somatosensory and Motor cortex

In the present study, an ipsilateral decrease in the synchrony of the resting activity of the somatosensory cortex was observed following 10 Hz LI-rTMS. Our finding is compatible with those of an fMRI study by Schneider, *et al*.^60^ showing an increase in activity of the targeted brain region when 5 Hz rTMS (considered to be excitatory and have roughly equivalent effects to 10 Hz^59^) was applied over the left primary somatosensory cortex.

In contrast, when comparing the spatial maps post-cTBS with the pre-stimulation group, there is a clear increase in the synchronised activity in the ipsilateral versus the contralateral somatosensory cortex (Fig. 6). This is in agreement with a study by Rai, *et al*.^61^ evaluating the effect of cTBS over the left somatosensory cortex by measuring the change in tactile acuity of the contralateral hand. They noted a decrease in neural activity within the stimulated cortex after cTBS application, which may indicate that cTBS can reduce sensory processing in the ipsilateral cortex. The increase in synchrony of resting-state activity in the present results and the localised decrease in activity in the ipsilateral somatosensory cortex following cTBS in Rai’s study appear to reflect similar trends, suggesting that cTBS effects on the somatosensory cortex may be restricted to the stimulated hemisphere and not spread bilaterally.

The effects of 1 Hz LI-rTMS are more variable across studies and may differ more between rodent LI-rTMS and human rTMS. While reports in humans suggest a purely uni-hemispheric effect of rTMS, the present results show bilateral and even contralateral outcomes following LI-rTMS. Enomoto, *et al*.^62^ examined the changes in excitability of the sensory cortex following rTMS by measuring changes in the somatosensory evoked potentials and found that 1 Hz rTMS over the left primary motor cortex suppressed the activity of only the ipsilateral sensory cortex. Similarly, Vidoni, *et al*.^63^ studied the impact of 1 Hz rTMS over left primary somatosensory cortex by observing cutaneous somatosensation and found that while the right hand was significantly impaired, the ipsilateral left hand was unaffected. In contrast, we found that the synchrony of the resting activity of the somatosensory cortex was decreased bilaterally following 1 Hz stimulation. Although we cannot rule out a lack of focality in the present study relative to human studies, some components nonetheless showed an exclusively contralateral decrease in synchrony, while those with bilateral decrease showed a stronger ipsilateral decrease in the synchrony of resting activity within the same component, suggesting that changes induced by 1 Hz rTMS are likely to be complex.

There is also evidence that 1 Hz rTMS in other brain regions has effects on the contralateral hemisphere, which is congruent with some of the results herein on synchrony of resting activity. 1 Hz, being an inhibitory frequency, is thought to decrease the activity of inhibitory neurones in the stimulated hemisphere, causing a reduction in the inhibitory interhemispheric drive, which in turn leads to an increase in excitability of the contralateral hemisphere. This effect of 1 Hz rTMS has been exploited in treating stroke patients by applying low-frequency stimulation to the unaffected hemisphere to decrease transcallosal inhibition of the lesioned hemisphere and consequently improve motor function^64–66^. That the motor cortex in both hemispheres experiences a change in neuronal excitability following 1 Hz rTMS on one hemisphere may explain the bilateral changes in synchrony observed in the present study. Interestingly, applying high-frequency rTMS to the lesioned hemisphere can have a similar effect by improving ipsilesional hemispheric excitability and hence improving motor rehabilitation. In a stroke study, 5 Hz (high-frequency) rTMS was applied ipsilesionally, and a bilateral increase in motor connectivity was found^20^. In accordance with this study, we also found a bilateral increase in the synchrony of resting activity in the motor cortex following 10 Hz stimulation.

Previous human studies have found that there are bilateral changes in the motor cortex activity following cTBS stimulatiom^67,68^. However, in the present study, only an ipsilateral increase in the synchronised activity of the motor cortex was observed following cTBS LI-rTMS to the right hemisphere of the rat brain. Although one can argue that there were also contralateral changes in synchrony of resting-state activity following cTBS (Fig. 6), these changes have not been reported because they did not encompass entire brain regions, as identified in the atlas, and similar spurious changes were found in the sham data. The intrinsic differences between the methods used to detect changes in correlation and activity or the limitations in imaging measurements like EPI distortions could be the cause of this inconsistency.

#### Hippocampus

While the proximal changes in the DMN may reflect direct stimulation of those brain regions, the very low intensity of the magnetic field applied in LI-rTMS (Fig. 5) means that any change in the activity of the hippocampus would likely be indirect and due to the modulation of functionally connected regions. We detected an ipsilateral decrease in the synchronised activity of the hippocampus following 10 Hz stimulation. This result is supported by Wang, *et al*.^69^ who applied high-frequency (20 Hz) stimulation to the left lateral parietal cortex of healthy adults to non-invasively enhance the targeted cortical-hippocampal networks and study their role in associative memory. An ipsilateral change in the hippocampus was detected following multiple-session stimulation and the increased functional connectivity was correlated with improved associative memory performance. Hence, the present results display a correlation profile that is coherent with what is known about the effect of high-frequency rTMS on the hippocampus in the literature.

After 1 Hz stimulation, we found a bilateral decrease in the synchrony of hippocampal activity relative to other brain regions. Van der Werf, *et al*.^70^ also determined that the hippocampus had reduced activation bilaterally following the application of low-frequency rTMS over the left dorsolateral prefrontal cortex. They hypothesised that this change was not due to direct stimulation because the changes in neural activity were observed distally relative to the site of stimulation. Consistent with this finding, the change in the synchronised activity of the hippocampus observed in the resting-state network in the present study could, therefore, be due to the change in cortical excitability or the transcallosal spread of LI-rTMS effects inducing bilateral inhibition as discussed above for motor and somatosensory cortices.

BHFS is a relatively new pattern of stimulation and use in humans has yet to be reported. As such, there is little information about the effects of BHFS in the literature. Studies using BHFS LI-rTMS in mice have shown increased structural plasticity of visual pathway topography in the midbrain, thalamus and cortex^23,24^ and altered density of GFAP astrocytes in a mouse model of brain injury^71^, possibly via intracellular calcium increases and changes in gene expression^27^. However, how these cellular and molecular changes might relate to resting-state network changes remains unclear. In the present study, like 1 Hz stimulation, BHFS had a bilateral effect on the synchronised activity in the somatosensory cortex and the hippocampus. Motor cortex resting activity following BHFS LI-rTMS showed a contralateral decrease in the synchrony compared to other brain regions, a different effect than observed with the other three LI-rTMS protocols. Further studies in animals and humans are warranted in effort to investigate the effects of BHFS on the resting-state networks.

### Use of anaesthetics in rs-fMRI and rTMS studies in rodents

Combined rTMS/rs-fMRI studies allow direct comparison between human and animal investigations, but these comparisons are complicated by the use of anaesthesia in animals. In human studies, the physiological condition of the subject can be assumed to be relatively constant throughout an rs-fMRI scan session^72^. In contrast, in animal rs-fMRI, the use of anaesthesia is required to immobilise the animal and reduce stress^72,73^.

However, the effects of anaesthetics may confound both imaging and rTMS experiments, as these neuroactive substances may cause alterations in neural activity, vascular reactivity and neurovascular coupling. Nonetheless, the DMN has been shown to persist irrespective of the depth and type of anaesthetics used^73–76^, and many rTMS studies using other (non-fMRI) methodologies have employed the use of anaesthetics (e.g., ketamine, pentobarbital, midazolam, isoflurane, propofol, and urethane) and have demonstrated the induction of neuronal plasticity in these anesthetised animals^77–79^, albeit with some impact of anaesthesia on rTMS outcomes^78,79^. Urethane, in particular, is commonly used in rTMS rodent electrophysiology studies because of its minimal effects on cortical excitability, its ability to preserve spinal reflexes and its capacity to maintain a stable resting motor threshold over an extended period^78,80^. However, urethane has mutagenic, carcinogenic, and hepatotoxic properties, which limit its use to acute and terminal experimental investigations. Longitudinal experiments in animals, such as those described here, therefore require alternative anaesthetic options.

In the present study, we have used isoflurane, even though studies indicate some concerns with its use in the context of both rTMS and fMRI imaging. Isoflurane may affect the intracellular concentration of calcium^81^, potentially modulating presynaptic transmission and/or postsynaptic excitability. Isoflurane also decreases excitatory and increases inhibitory transmission, causing an overall suppression of neural activity^81,82^. As such, in the presence of isoflurane, the ability of high-frequency rTMS to depolarise is impaired^79^. Additionally, isoflurane, being a GABAergic anaesthetic, induces vasodilation^83^, particularly in deep anaesthesia, through the activation of ATP-sensitive potassium channels of smooth muscle cells in cerebral arteries^72^. Vasodilation leads to an increase in cerebral blood flow, which may be interpreted as an increase in activity. Despite these confounding factors, isoflurane is the anaesthetic of choice for repeated long-term experiments because of its ease of use and control, and rapid reversibility^84^. Isoflurane level can be kept within a specified range (1–2.5% in the present study) within and between experiment sessions. The concentration of isoflurane can also be adjusted (within the specified range: 1–2.5%) based on the monitoring to keep the physiological parameters from fluctuating outside the desired range. The lack of change in synchronised resting activity observed after sham stimulation in our study provides confidence that the experimental conditions were stable over time and within and between individuals. Moreover, the reproducibility maps (Figs. 4A and B) show that the correlation at the centre of a cluster was always greater than a z-score of 2 irrespective of the timepoint and animal, suggesting that our results have biological significance.

### Conclusion

To date, all reported studies on the effects of rTMS on the structure and function of the DMN have been conducted in humans. To the best of our knowledge, the present study is the first to show evidence of alterations in the resting-state networks caused by LI-rTMS in a pre-clinical model and most of the observed changes are consistent with those described in the human rTMS literature. Nonetheless, the precise mechanisms generating these changes in resting neuronal activity remain to be elucidated. Furthermore, rTMS and LI-rTMS may have similar impact on the DMN of humans and animals, despite significant differences in intensity and focality of stimulation. To better understand the mechanisms underlying the reported clinical benefits of rTMS in different neurological and psychiatric conditions, relevant animal models could be used to link the LI-rTMS-induced changes in resting brain activity to changes in symptoms (through behavioural tests). Subsequent invasive techniques such as molecular studies can then be used to explore those effects in greater detail and provide information about how observed functional changes reflect those detected at a molecular and cellular level. This study provides a framework to use brain imaging to explore how LI-rTMS affects rodent resting brain activity, promoting evidence-based translation to human treatments.

## Electronic supplementary material

Supplementary information
