# Supplementary material for: Resting-state fMRI study of brain activation using low-intensity repetitive transcranial magnetic stimulation in rats
Source: Sci Rep. 2018 Apr 30;8:6706. doi: 10.1038/s41598-018-24951-6 (PMC5928106; doi:10.1038/s41598-018-24951-6)
Supplement: Supplementary file 1 — Supplementary information [file 41598_2018_24951_MOESM1_ESM.pdf]

Resting-state fMRI study of brain activation using low-intensity repetitive transcranial magnetic stimulation in rats

Bhedita J Seewoo[1,2,3], Kirk W Feindel[2,4], Sarah J Etherington[3], Jennifer Rodger[1,5*]

[1] Experimental and Regenerative Neurosciences, School of Biological Sciences, The University of Western Australia, Perth, WA, Australia
[2] Centre for Microscopy, Characterisation and Analysis, Research Infrastructure Centres, The University of Western Australia, Perth, WA, Australia
[3] School of Veterinary and Life Sciences, Murdoch University, Perth, WA, Australia
[4] School of Biomedical Sciences, The University of Western Australia, Perth, WA, Australia,
[5] Brain Plasticity Group, Perron Institute for Neurological and Translational Research, Perth, WA, Australia

*** Corresponding author:**
Associate Professor Jennifer Rodger
Experimental and Regenerative Neurosciences
School of Biological Sciences M317
The University of Western Australia,
35 Stirling Highway, Crawley WA 6009
Tel: + 61 (0) 8 6488 2245
Email: jennifer.rodger@uwa.edu.au
Mobile: 0411 844 103

Supplementary Table S1

Supplementary Table 1. Total number of pulses delivered during 10 min LI-rTMS at the different frequencies used. Adapted from Grehl et al.[26]

Frequency	Description	Total number of pulses delivered
**0 Hz (sham)**	Coil not activated	0
**1 Hz**	1 pulse per second	600
**10 Hz**	10 pulses per second	6,000
**cTBS**	3 pulses at 50 Hz, repeated at 5 Hz (Cárdenas-Morales *et al.*, 2010)	9,000
**BHFS**	62.6 ms trains of 20 pulses, repeated at 9.75 Hz (Grehl *et al.*, 2015)	120,000

Supplementary Figure S1

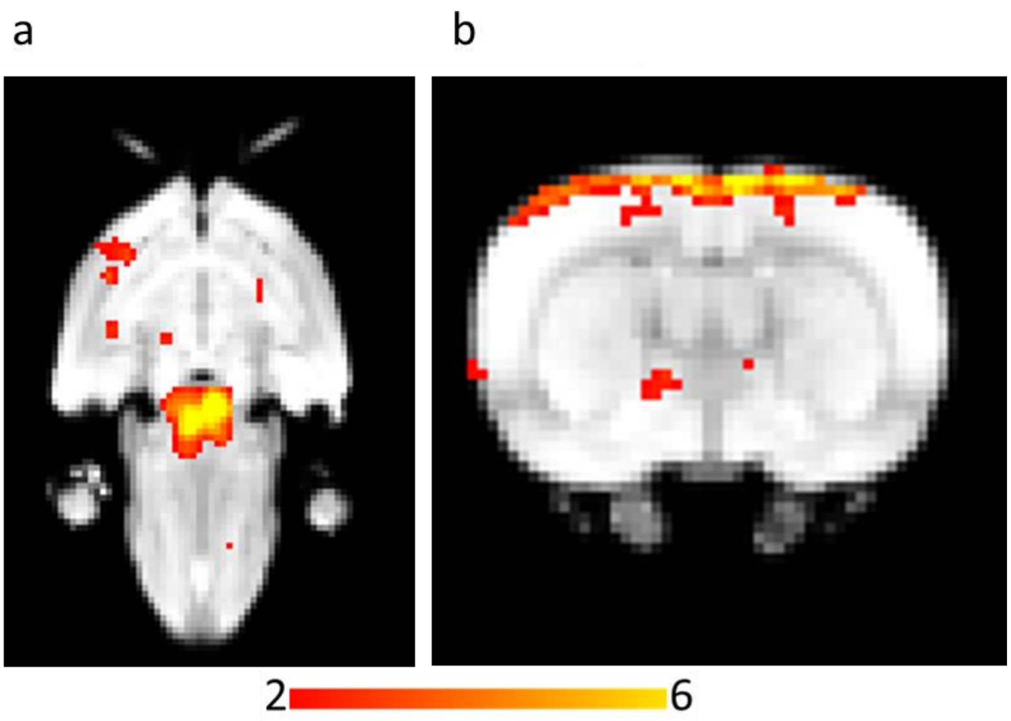

Supplementary Figure S1. Two independent components — one axial slice (a) and one coronal slice (b) — from group-ICA classified as 'noise' components. A shows highly synchronised resting activity in the brainstem while B shows highly synchronised activity only at the dorsal part of the coronal slice. Colour bar indicates z-scores (thresholded at z > 2, uncorrected p-value < 0.0455 for a two-tailed hypothesis).

Supplementary Table S2

Supplementary Table 2. Summary of changes in the default mode network in brain regions post-rTMS using 10 Hz and BHFS excitatory frequencies and 1 Hz and cTBS inhibitory frequencies on the right (R) side (ipsilateral changes) and on the left (L) side (contralateral changes) of the brain. The numbers in brackets correspond to the numbers used to denote the different brain regions in Figure 5. '↑' refers to an increase in correlated activity, '↓' refers to a decrease in correlated activity and '-' means no change.

Region	Function	Excitatory frequencies				Inhibitory frequencies			
		10 Hz		BHFS		1 Hz		cTBS	
		R	L	R	L	R	L	R	L
**Orbital cortex (1)**	Decision making and emotional processing	↑	↑	-	-	↑	↑	↑	-
**Auditory cortex (3)**	Hearing functions	↓	-	↓	↓	-	↓	-	↓
**Somatosensory cortex* (4)**	Receives all sensory input	↓	-	↓	↓	↓	↓	↑	-
**Striatum (5)**	Facilitates voluntary movement	↓	-	↓	-	↓	-	↑	↑
**Retrosplenial cortex (6)**	Episodic memory and spatial navigation	↓	-	-	-	↓	↓	-	-
**Entorhinal cortex (10)**	Learning and memory	↓	-	↓	-	-	-	-	-
**Hippocampus* (11)**	Regulates emotions, spatial learning and memory	↓	-	↓	↓	↓	↓	-	-
**Visual cortex (12)**	Receives and processes visual input	-	-	-	↓	-	↓	-	-
**Inferior colliculus (13)**	Part of the auditory system	↓	-	↓	-	-	-	-	-
**Motor cortex* (14)**	Motor function and motor planning	↓↑	↑	-	↓	↑	↓	↑	-

*These brain regions are the primary targets of stimulation.
